# Epigenetic regulation of NR5A2 influences colorectal cancer cell stemness via a stemness-related transcription factor NANOG

**DOI:** 10.1186/s12964-025-02477-5

**Published:** 2025-11-10

**Authors:** Jia Liu, Li Li, Liang Zhang, Wenpeng Wang, Lei Zheng, Dalu Kong, Jiefu Wang, Yang Zhan

**Affiliations:** 1 Department of Colorectal Cancer, Tianjin Medical University Cancer Institute and Hospital, National Clinical Research Center for Cancer, Tianjin’s Clinical Research Center for Cancer, Key Laboratory of Cancer Prevention and Therapy, Tianjin Key Laboratory of Digestive Cancer, Tianjin, 300060 China; 2 Department of Gynecologic, NHC Key Laboratory of Hormones and Development, Tianjin Key Laboratory of Metabolic Diseases, Chu Hsien-I Memorial Hospital & Tianjin Institute of Endocrinology, Tianjin, 300134 China; 3 Department of surgical oncology, Jiuquan Second Peoples Hospital, Jiuquan, 735000 China

**Keywords:** NR5A2, Colorectal cancer, Cancer stem cell, NANOG, Epigenetic regulation

## Abstract

**Objective:**

Colorectal cancer (CRC) is an aggressive malignancy with high mortality, and the identification of upstream regulators of stemness represents a critical step toward developing more effective targeted therapies. This study aimed to define the role of NR5A2 in CRC, particularly in the context of cancer stem cells (CSCs).

**Methods:**

We profiled DNA copy number alterations, DNA methylation status, and mRNA expression in 30 CRC specimens. Primary CRC cells and CSC-enriched sphere cultures were established in vitro. Functional assays included RNA sequencing, sphere- and colony-formation assays, cell viability and cytotoxicity assessments, qRT-PCR, western blotting, immunofluorescence staining, chromatin immunoprecipitation (ChIP), and in vivo patient-derived xenograft (PDX) models.

**Results:**

Integrated genomic and epigenomic analyses implicated NR5A2 in the progression of an aggressive CRC subtype characterized by stemness gene expression. Our findings demonstrate that NR5A2 plays a key role in CRC. Mechanistically, NR5A2 promoted CSC maintenance by directly binding to the promoter and enhancer regions of NANOG, thereby upregulating its expression. Pharmacological inhibition of NR5A2 using Cmp3 significantly sensitized a subset of CRC PDX models to standard chemotherapy, resulting in enhanced tumor regression.

**Conclusion:**

This study identifies NR5A2 as a novel, actionable therapeutic target in CRC. Pharmacological modulation of NR5A2 disrupts CSC-driven stemness, potentially preventing relapse and improving treatment outcomes. These findings provide a strong rationale for the development of NR5A2-targeted therapies, either as monotherapy or in combination with chemotherapy, to optimize CRC patient care.

**Supplementary Information:**

The online version contains supplementary material available at 10.1186/s12964-025-02477-5.

## Highlights

1. NR5A2 was identified as a key therapeutic target in colorectal cancer (CRC) through a dual mechanism involving the regulation of NANOG.

2. A significant proportion of CRC tumors demonstrated responsiveness to NR5A2 inhibition, particularly when combined with tumor-debulking chemotherapy.

3. These findings support NR5A2-targeted therapy as a promising strategy to improve clinical outcomes in CRC patients.

**Supplementary Information:**

The online version contains supplementary material available at 10.1186/s12964-025-02477-5.

## Introduction

Within the global landscape of diseases, Colorectal cancer (CRC) represents a substantial burden and is categorized among the prominent malignant neoplasms that pose severe threats to human health and survival [1, 2]. In 2023 alone, approximately 153,020 new CRC cases and 52,550 CRC-related deaths were projected in the United States, including 19,550 cases and 3,750 deaths among individuals under 50 years of age [3]. In spite of persistent reductions in the overall occurrence rate, CRC currently exhibits a growing prevalence among younger individuals, is diagnosed at more advanced pathological stages, and emerges more frequently in the left colon and rectum. Systematic patterns of epigenomic and genomic disruption in these disorders have recently been elucidated through large-scale, multi-omics investigations of malignancies. Frequent genomic aberrations, prompted by DNA copy number alterations or mutations, occur throughout tumor development and facilitate disease advancement. Epigenetic regulation through DNA methylation critically shapes heterogeneous cancer phenotypes. Specifically, within CRC, genomic analyses highlight the marked diversity in epigenomic and genomic alterations [4, 5]. DNA copy number variations exhibit critical regulatory functions in CRC pathogenic progression; furthermore, transcriptional dysfunctional expression resulting from these aberrant changes represents a plausible driver mechanism for disease progression [6–8]. DNA methylation profilometric analyses further demonstrate the biological as well as clinical relevance of epigenetic control in CRC evolution [9–11]. Nevertheless, while DNA copy number changes and methylation patterns exert genome-spanning effects in malignancies, whether DNA copy number alterations mechanistically interrelate with epigenetic DNA methylation patterns, or whether such interactive mechanisms facilitate the development of oncogenesis, remains undetermined. To address this gap, we characterized DNA copy number profiles, DNA methylation patterns, and messenger RNA (mRNA) expression levels in a cohort of CRC patients.

Emerging evidence indicates that cancer stem cells (CSCs)—a subpopulation of tumor cells with unlimited self-renewal and multipotent differentiation capacities—play a critical role in CRC initiation, progression, metastasis, and resistance to therapy [12–14]. Crucially, within aggressive and therapy-resistant malignancies, a distinct CSC subset survives initial treatment efficacy and drives tumor recurrence through heightened invasive properties and reduced therapeutic sensitivity [15, 16]. This paradigm underscores the imperative to devise novel CSC-directed therapeutic interventions for achieving durable remissions.

Liver receptor homolog 1 (nuclear receptor LRH-1, NR5A2), a ligand-responsive transcription factor, exhibits pleiotropic roles in development and pathogenesis [17]. During embryogenesis, NR5A2 orchestrates the differentiation of critical organ systems, including the liver, intestine, and pancreas [18, 19]. Simultaneously, NR5A2 maintains the pluripotent state of stem cells and regulates key pluripotency-associated factors such as Oct4 and Nanog [20, 21]. Based on this biology, we conducted a comprehensive investigation to elucidate the phenotype-specific roles of NR5A2 in CRC progression and to evaluate its potential as a therapeutic target. Furthermore, we assessed the effects of pharmacological inhibition using the selective small-molecule inhibitor Cmp3, which binds to the ligand-binding domain of NR5A2 and attenuates its transcriptional activity [22]. In this study, we explored the ramifications of pharmacological inhibition by Cmp3 on NR5A2. By targeting NR5A2, our objective was to disrupt the regulatory circuitry governing cancer stemness and evaluate its translational applicability for the clinical management of CRC.

## Method

### Profiling of DNA copy numbers, DNA methylation, and mRNA expression

#### Clinical sample collection

CRC tissue samples and paired adjacent normal samples were obtained from 30 patients at Tianjin Medical University Cancer Institute and Hospital, with informed consent got from participants (or their families at post-mortem exams). The specimens were snap-frozen in liquid nitrogen and stored at − 80 °C as previously described until used. The frozen tissues from 30 cases of tumor specimens and 30 cases of the non-tumoral surrounding tissue specimens were used for DNA copy-number variation (CNV), DNA methylation (MET), and mRNA expression (EXP) profiling. Detailed methods have been shown in Supplementary Materials.

#### Culture and treatment of primary human CRC cells

Patient-derived tumor xenograft (PDX) tissues, grown subcutaneously in the flank of immunodeficient BALB/c nude mice (Charles River), were used to isolate primary CRC cells with 2% collagenase P (Roche) and 1 mg/mL dispase (Life Technologies). Minced fragments of PDX-derived tumor tissues were digested with Collagenase for 1.5 h at 37 °C. Cells were cultured in RPMI media supplemented with 10% FBS and 50 units/ml penicillin/streptomycin. A final concentration of 10 nM of siRNA targeting the NR5A2 gene and negative control were used to transfect the primary CRC cells with HiPerFect transfection reagent (QIAGEN). The siRNA sequences are provided in Supplementary Table S1. The NR5A2 inhibitor Cmp3 (PC-21458, ProbeChem) was dissolved in DMSO at 10 mM stock concentration and applied to the desired final concentrations in cell cultures.

#### CSC-enriching sphere cultures

Primary CRC cell suspensions were cultured for seven days in ultra-low attachment plates (Corning) at 10,000 cells/mL density in DMEM-F12 (Invitrogen) supplemented with B-27 (GIBCO) and bFGF (PeproTech EC). For serial passaging, termed 2nd generation sphere formation, 7-day spheres were retained using a 40 μm cell strainer, dissociated into single cells with trypsin, and reseeded at 10,000 cells/ml in the same conditions for another seven days. The number of primary and secondary spheres was determined using a CASY cell counter (Roche).

#### In vivo PDX model

Primary CRC cells (1 × 10^5^ in 50 µl Matrigel™) were injected subcutaneously or were implanted orthotopically into 6–8-week-old BALB/c nude mice (Charles River). Once CRC tumors had reached ~ 0.2 cm^3^, mice were randomized to vehicle control, Cmp3 alone, Oxaliplatin alone, or Oxaliplatin plus Cmp3.

### Statistical analysis

GraphPad Prism 6.0 was used to perform statistical analyses. All values are expressed as means ± SD and the Bonferroni correction method was applied to correct multiple testing to control the false discovery rate (FDR). A two-tailed Student’s t-test was used to determine the statistical significance of differences between experimental groups. *P* < 0.05 was considered to be statistically significant.

## Results

### Transcriptome deregulation by DNA copy number and methylation

We first investigated whether expression profiles of DNA copy number–correlated (CNVcor) and DNA methylation–correlated (METcor) genes could stratify prognostic subgroups. Kaplan–Meier survival analyses demonstrated that CNVcor- and METcor-defined subtypes robustly predicted patient outcomes (Figure S1).

**Fig. 1:**
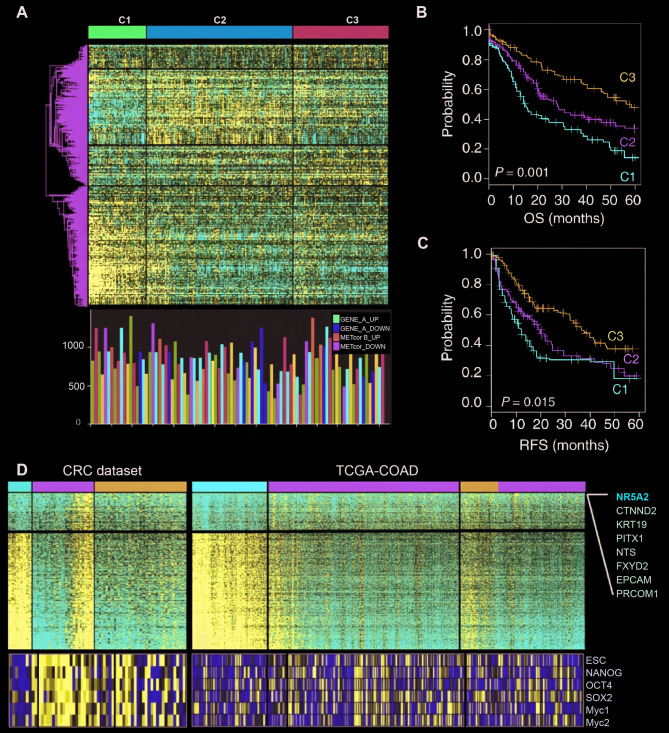
Transcriptome deregulation by DNA copy number or methylation. **A** Heatmap displaying expression profiles of differentially expressed genes across CRC subtypes identified by iClusterPlus analysis (top). Frequencies of CNVcor_UP, CNVcor_DOWN, METcor_UP, and METcor_DOWN genes are shown (bottom) **B**, **C** Kaplan–Meier survival curves illustrating overall survival (OS) **B** and recurrence-free survival (RFS) **C** among the identified CRC subtypes. **D** Heatmaps of commonly upregulated (DEG_UP, *n* = 121) and downregulated (DEG_DOWN, *n* = 366) genes shared between CRC and TCGA-COAD datasets (upper). Enrichment of stemness gene sets (ESC, Nanog, Oct4, Sox2, Myc1, and Myc2) in CRC and TCGA-COAD datasets is presented (lower). Top-ranked DEGs are annotated

To further delineate clinically meaningful molecular subtypes, we integrated DNA copy number, DNA methylation, and RNA expression data using a unified clustering approach. This analysis identified three distinct subtypes (C1–C3) characterized by striking differences in CNV and methylation aberration frequencies (Fig. 1A). Kaplan–Meier analyses revealed significantly different survival outcomes across subtypes: patients with C1 tumors exhibited the poorest overall survival (OS) and recurrence-free survival (RFS), whereas those with C3 tumors had the most favorable prognosis (Fig. 1B, C). These results validate the prognostic utility of the CNVcor-METcor classification system for identifying clinically distinct CRC subgroups with unique genomic and epigenomic dysregulation.

To define transcriptomic drivers of these subtypes, we identified differentially expressed genes (DEGs) shared between our cohort and TCGA-COAD datasets. Notably, NR5A2 emerged as the most significantly upregulated gene in the C1 subtype (Fig. 

1D). Aggressive C1 tumors also displayed elevated expression of stemness-associated genes such as KRT19, EPCAM, and PROM1. Enrichment analysis of stemness gene sets (ESC, Nanog, Oct4, Sox2, Myc1, and Myc2) further confirmed significant upregulation in C1 tumors. These findings suggest that NR5A2 may contribute to the aggressive, stemness-enriched phenotype of this CRC subtype.

### NR5A2 regulates stemness in CRC

**Fig. 2:**
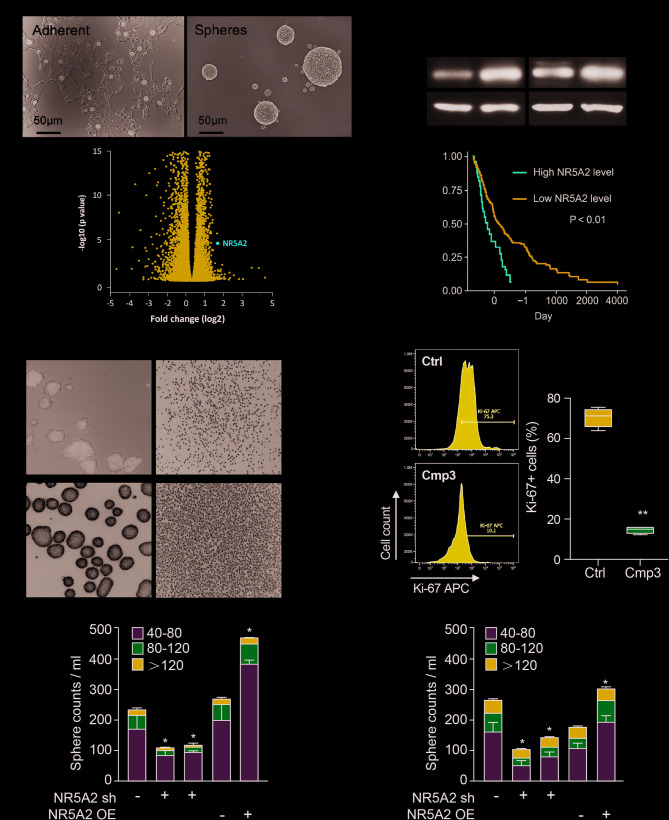
NR5A2 regulates stemness in CRC. **A** Upregulation of stemness-associated mRNAs in CSC-enriched sphere (Sph) cultures compared with adherent (Adh) cultures derived from primary CRC tumors. Representative images of adherent and sphere cultures are shown (top), and RNA-seq fold changes for NR5A2 expression are displayed (bottom). **B **Western blot analysis comparing NR5A2 protein levels in Adh versus Sph cultures from CRC patient-derived xenografts, with β-actin as loading control. **C **Kaplan–Meier curve showing the prognostic significance of NR5A2 mRNA expression for overall survival in CRC patients. **D** Representative images of sphere formation on day 7 following Cmp3 treatment (60 µM). **E** Flow cytometric analysis of Ki-67 expression after 72 h of Cmp3 treatment (60 µM) **F** Secondary sphere-forming capacity following NR5A2 knockdown (sh) or overexpression (OE). * *p* < 0.05, ** *p* < 0.01, *** *p* < 0.001

To explore the functional role of NR5A2, we performed RNA sequencing (RNA-seq) on CSC-enriched anchorage-independent sphere cultures compared with adherent cultures. NR5A2 was among the most significantly upregulated genes (Fig. 2A). Consistent with RNA-seq results, NR5A2 expression was increased following sphere culture (Fig. 2B). tratification of CRC patients by NR5A2 expression levels revealed that high NR5A2 expression correlated with significantly reduced OS (Fig. 2C), supporting its association with poor prognosis.

We next assessed the functional effects of NR5A2 inhibition on colorectal CSCs. Forming spheres were treated with Cmp3 every 48 h, which led to a pronounced suppression of 3D sphere formation, significantly decreasing both the number and size of spheres compared with controls (Fig. 2D; Fig. S2A). Caspase-3/7 immunofluorescence (Fig. S2B) nd Annexin V flow cytometry (Fig. S2C) emonstrated no increase in apoptosis within the relevant pharmacological range of Cmp3 concentrations after 72 h. In contrast, flow cytometric analysis revealed a dose-dependent reduction in cell proliferation, as indicated by Ki-67 staining (Fig. 2E; Fig. S2D). Together, these findings indicate that NR5A2 inhibition primarily suppresses proliferative capacity in differentiated CRC cells rather than inducing apoptosis.

To corroborate these results, we employed genetic strategies to modulate NR5A2 expression and evaluated their effects on secondary sphere formation. After primary spheres were generated by day 7, spheres larger than 40 μm were collected, dissociated into single-cell suspensions, and re-cultured for 7 days to enrich for cells with stem-like properties. NR5A2 knockdown consistently diminished secondary sphere-forming capacity, whereas NR5A2 overexpression enhanced it (Fig. 2F). Consistent with this, Cmp3 treatment inhibited both primary and secondary sphere formation, confirming its ability to suppress CSC self-renewal. Although MAA failed to inhibit primary sphere formation at 40 µM (Fig. S2D&E), it significantly impaired secondary sphere formation, further supporting that NR5A2 inhibition selectively targets CSC functionality at relatively low concentrations.

**Fig. 3:**
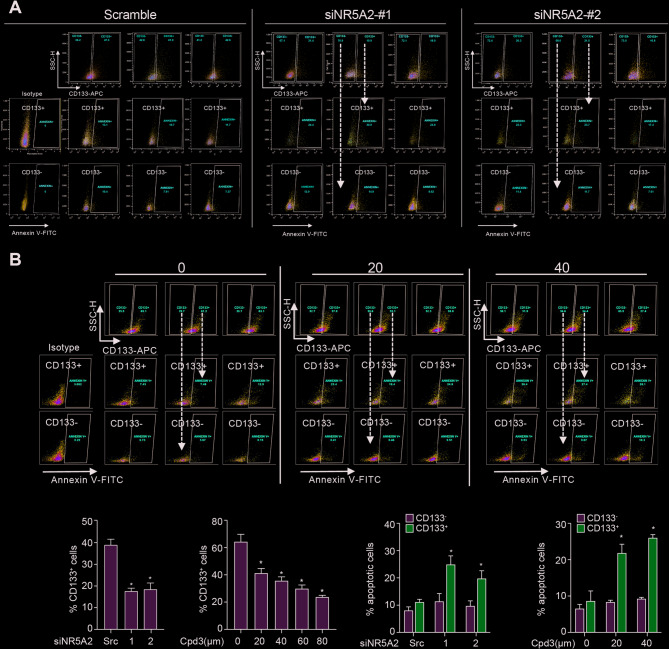
Inhibition of NR5A2 specifically eliminates tumor-initiating CSCs. **A**, **B** Flow cytometric analysis of Annexin V staining in second-generation spheres sorted into CD133⁺ and CD133⁻ cells and treated with two distinct siNR5A2 variants or Cmp3 (20 µM or 40 µM). **C**, **D** Quantification of CD133⁺ CSC populations after genetic or pharmacologic NR5A2 inhibition. **E**, **F** Proportion of Annexin V–positive apoptotic cells among CD133⁺ CSCs (blue) and CD133⁻ CRC cells (grey). *p < 0.05, **p < 0.01, ***p < 0.001

To further investigate the dual role of NR5A2 in CRC, we examined its specific function within the CSC compartment (Fig. 3A, B; Fig. S3). Second-generation spheres, which are highly enriched for CSCs, were treated with siNR5A2 for 72 h, and CD133⁺ CSC levels were quantified. siNR5A2 treatment resulted in a pronounced reduction in the CD133⁺ population. As depicted in Fig. 3C, treatment with siNR5A2 resulted in a decrease in CD133^+^ CSCs. These findings prompted us to determine whether pharmacological inhibition of NR5A2 would yield comparable results. Treatment with graded doses of Cmp3 (20–80 µM) produced a dose-dependent reduction in the CD133⁺ CSC population (Fig. 3D). Notably, this decrease was accompanied by a robust increase in Annexin V staining, indicating selective induction of apoptosis within the CD133⁺ CSC population. Both Cmp3 treatment and siNR5A2 transfection reproduced this apoptotic phenotype (Fig. 3E, F). In contrast, differentiated CD133⁻ CRC cells exhibited minimal Annexin V positivity, underscoring the CSC-specific nature of NR5A2 inhibition–induced apoptosis.

### NR5A2 promotes stemness via upregulating NANOG

**Fig. 4:**
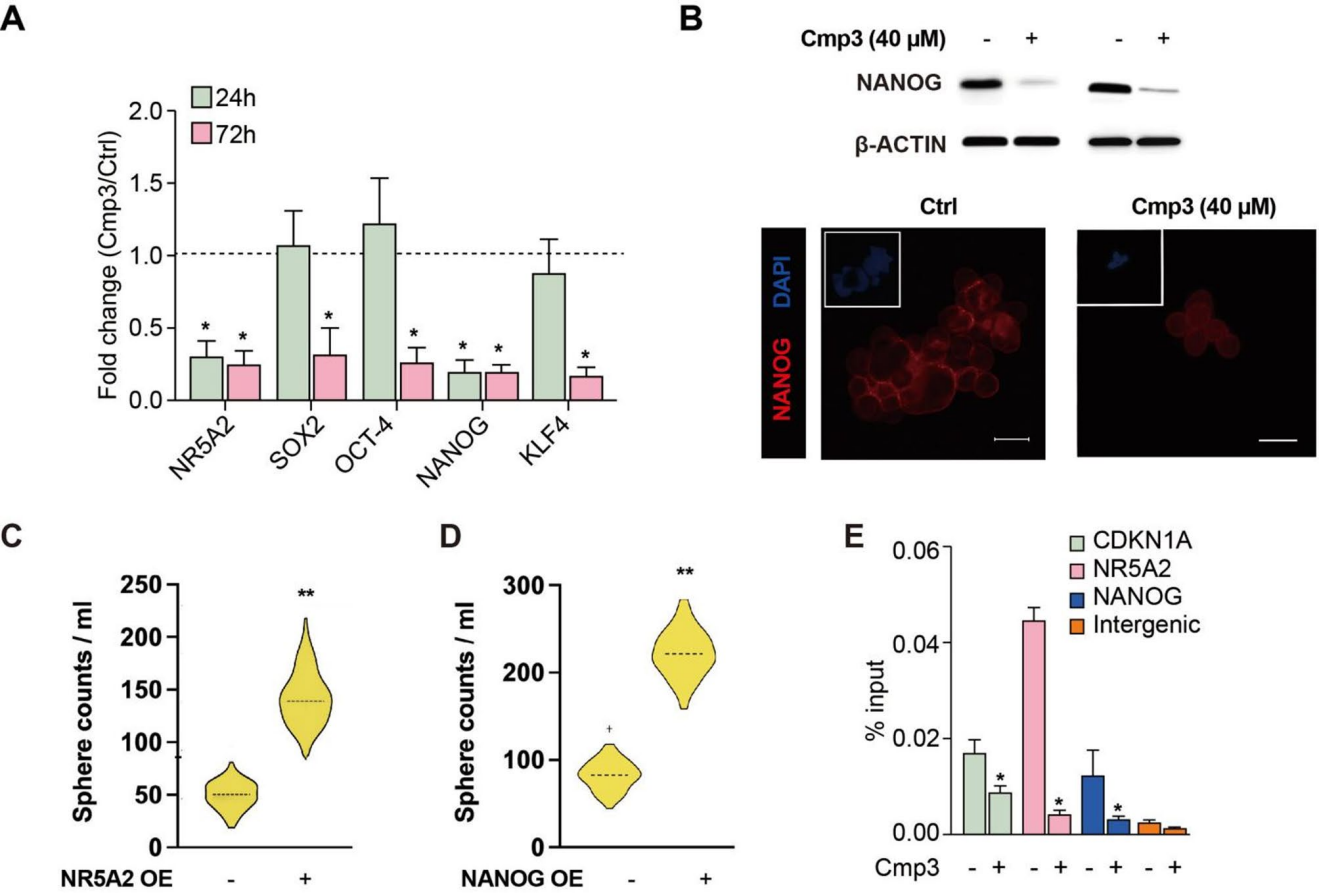
**A **NR5A2 promotes stemness via upregulating NANOG expression. CSC frequency following 7 days of treatment, defined as CD133⁺CD44⁺ or CD133⁺CXCR4⁺ populations as assessed by flow cytometry. **B** Western blot and immunofluorescence analyses of NANOG protein levels in CRC patient-derived CSC cells following DMSO or Cpd3 treatment (40 µM) for 24 h. **C** Sphere formation capacity using sorted CD133 + CRC cells following overexpression of NR5A2 or NANOG. The dashed lines in the violin plots indicate the median.* *p* < 0.05, ** *p* < 0.01, *** *p* < 0.001

To elucidate the mechanism by which NR5A2 promotes stemness in CRC, we profiled the expression of stemness-associated transcription factors following Cmp3 treatment. Quantitative PCR performed 24 h after NR5A2 inhibition revealed a significant downregulation of NANOG (Fig. 4A; Fig. S4A), whereas SOX2, OCT4, and KLF4 remained unchanged at this early time point. Notably, decreases in SOX2, OCT4, and KLF4 expression were observed only after 72 h of treatment. Western blot and immunofluorescence analyses confirmed a corresponding reduction (not enhancement) of NANOG protein levels at 24 h (Fig. 4B), consistent with transcriptional downregulation. Functionally, NR5A2 overexpression enhanced sphere formation (Fig. 4C), and a similar effect was observed upon direct overexpression of NANOG (Fig. 4D), implicating NANOG as a critical downstream effector of NR5A2. To further validate this regulatory axis, we performed a rescue experiment in which NANOG expression was reintroduced following Cmp3 treatment. Overexpression of NANOG successfully restored sphere-forming capacity in CRC cells despite pharmacologic inhibition of NR5A2 (Fig. S4B), confirming that NANOG mediates the pro-stemness effects of NR5A2. Collectively, these findings demonstrate that NR5A2 sustains CSC stemness in CRC through transcriptional upregulation of NANOG and that this pathway can be effectively targeted by Cmp3.

To determine whether NR5A2 directly regulates NANOG transcription, we performed chromatin immunoprecipitation (ChIP) assays targeting the NANOG promoter. Significant NR5A2 binding was observed at two promoter/enhancer sites compared with a negative intergenic region, with binding levels comparable to those at the CDKN1A enhancer (Fig. 4E). Notably, Cmp3 treatment nearly abolished NR5A2 occupancy at these sites, demonstrating that pharmacological inhibition disrupts NR5A2-mediated NANOG transcriptional activation. Collectively, these results establish that NR5A2 directly upregulates NANOG expression, thereby sustaining CSC stemness in CRC, and that this regulatory axis can be effectively targeted by Cmp3.

### NR5A2 Inhibition targets CSC in vivo

**Fig. 5:**
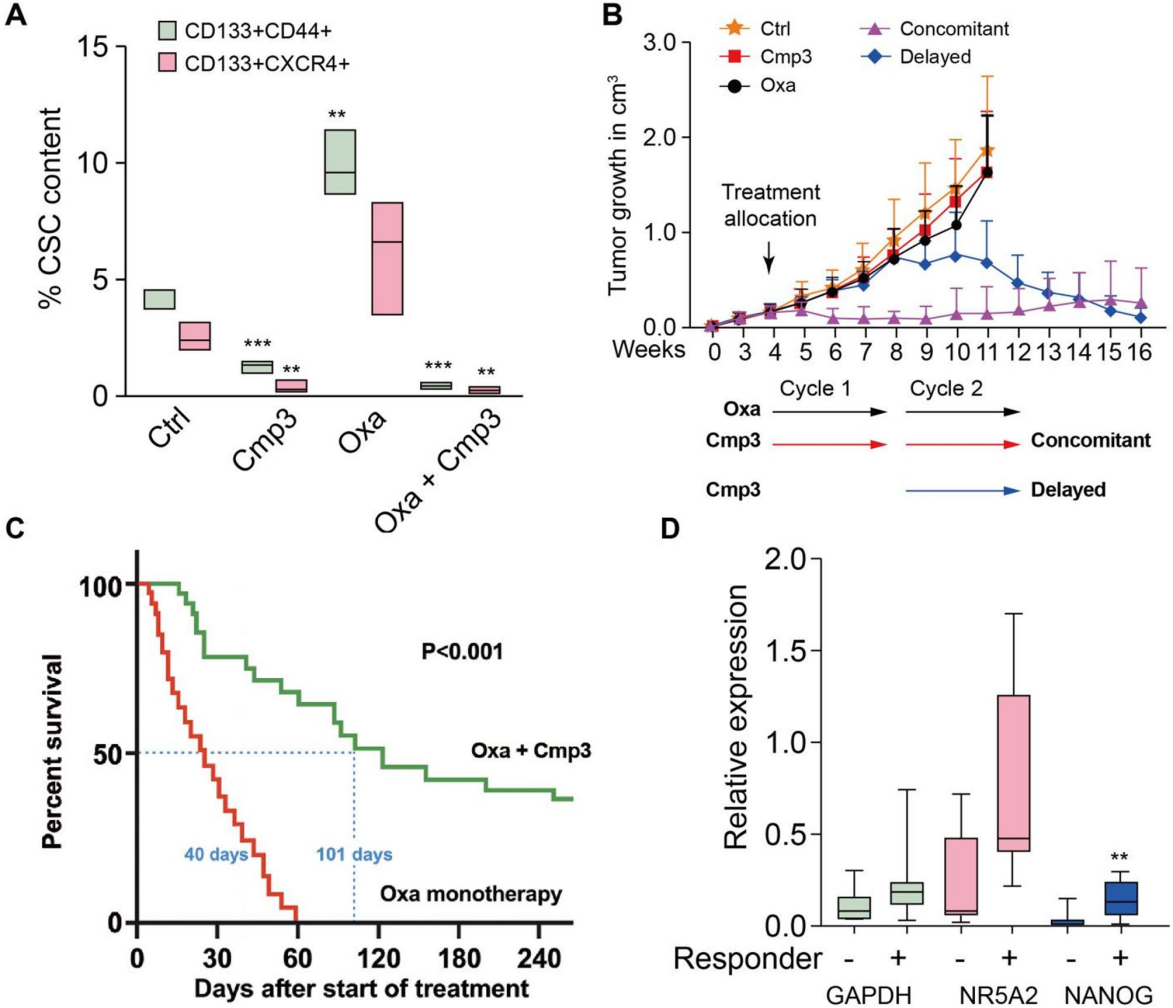
NR5A2 inhibition targets CSC in vivo. **A** CSC frequency following 7 days of treatment, defined as CD133⁺CD44⁺ or CD133⁺CXCR4⁺ populations as assessed by flow cytometry. **B** Tumor growth curves (cm³) following two 28-day treatment cycles with vehicle, Cmp3, oxaliplatin, or oxaliplatin + Cmp3. **C** Kaplan–Meier survival curves showing overall survival of tumor-bearing mice after treatment. **D** RT-qPCR analysis of baseline NR5A2 and NANOG mRNA levels. Responder (better OS) and non-responder (worse OS) of tumor-bearing mice the Oxa + Cmp3 group. * *p* < 0.05, ** *p* < 0.01, *** *p* < 0.001

These findings suggest that NR5A2 inhibition represents a promising therapeutic strategy to target CSCs in CRC. To assess its translational potential, we conducted in vivo intervention studies using a patient-derived xenograft (PDX) model classified as an intermediate responder to Cmp3. Once tumors reached ~ 0.2 cm³, mice were randomized to receive vehicle, Cmp3 alone, oxaliplatin alone, or oxaliplatin combined with Cmp3. In the combination arms, Cmp3 was administered either concurrently with oxaliplatin during cycles 1 and 2 (early combination) or initiated only during cycle 2 (delayed combination). CSC content was assessed at the end of the first cycle (day 28). Cmp3 monotherapy significantly reduced CSCs, defined as CD133⁺CD44⁺ or CD133⁺CXCR4⁺ populations, whereas oxaliplatin monotherapy paradoxically increased CSC frequency (Fig. 5A). Strikingly, the concurrent combination of oxaliplatin and Cmp3 nearly eliminated CSCs to undetectable levels. Extreme limiting dilution analysis demonstrated that the combination of oxaliplatin and Cmp3 markedly reduced CSC frequency, as evidenced by diminished secondary tumor formation in vivo (Fig. S5A).

Although Cmp3 monotherapy did not significantly suppress tumor growth (Fig. 5B), combined therapy induced rapid and durable tumor regression with no evidence of relapse over 12 weeks. (Fig. S5B). Even when Cmp3 administration was delayed until the second treatment cycle—at which point tumors had reached a larger volume—substantial and durable regression was still achieved.

Survival analyses further highlighted the therapeutic advantage of the combination approach: median survival was tripled in the oxaliplatin + Cmp3 group (99 days) compared with oxaliplatin alone (35 days) (Fig. 5C). Remarkably, 33% of mice treated with the combination regimen remained tumor-free through the entire 240-day follow-up. Correlation of baseline mRNA levels for NR5A2 and NANOG with treatment outcomes revealed a highly significant separation between responders and non-responders (Fig. 5D), suggesting that these biomarkers may serve as predictive indicators of therapeutic efficacy.

## Discussion

Through systematic investigation of colorectal cancer (CRC) pathogenesis, this study yielded pivotal findings that advance comprehension of disease mechanisms and therapeutic development. Prior research established that integrating multi-omics cancer genomic features enables molecular subtyping [23–25], unveiling the mechanisms of tumor heterogeneity while identifying therapeutic targets and biomarkers remains a central goal in cancer research. However, deciphering the immense complexity of cancer genomic datasets continues to pose significant challenges. The diverse etiologies and molecular landscapes of CRC further complicate the extraction of biologically meaningful insights from genomic information. To address this limitation, we systematically characterized the genomic and epigenomic regulators of copy-number variation–correlated (CNVcor) and methylation-correlated (METcor) genes. Our analyses demonstrated that these regulatory signatures enable robust classification of CRC into subtypes with distinct multi-omics profiles and divergent prognostic trajectories. These classifications retained validity in the heterogeneous TCGA-COAD cohort. Critically, NR5A2 exhibited hypermethylation and overexpression in CRC tissues and potentially drives progression of stemness-expressing aggressive subtypes.

Our results confirm NR5A2’s essential function as a stemness regulator in CRC. Within CRC stem cells, NR5A2 amplifies the activity of the stemness-associated transcription factor NANOG. Pharmacological and genetic modulation of NR5A2 expression enabled controlled suppression or induction of stemness properties. In PDX model investigations, NR5A2 inhibition significantly prolonged survival. Strikingly, combinatorial therapy with Oxaliplatin and Cmp3 reduced cancer stem cells (CSCs) to undetectable tumor levels, thereby optimizing therapeutic efficacy.

Mechanistically, we establish that NR5A2 transcriptionally activates NANOG to potentiate stemness in colorectal cancer (CRC) cells—a previously unreported regulatory axis. NANOG, a well-characterized core pluripotency transcription factor, is essential for maintaining stem cell self-renewal, facilitating cellular reprogramming, and preserving homeostatic equilibrium [26, 27]. An intricate regulatory network precisely controls NANOG cellular abundance at transcriptional, post-transcriptional, and post-translational tiers [28]. Ectopic NANOG expression occurs frequently in human CRC tumors and exhibits pronounced enrichment within cancer stem cells (CSCs) [29, 30]. Small interfering RNA-mediated NANOG silencing significantly impaired CSC invasive capacity [30], whereas lentiviral NANOG overexpression enhanced CRC cell motility, migration, and proliferation [31]. Notably, NANOG depletion sufficiently diminishes the CSC pool, confirming its functional centrality in this subpopulation [32]. Thus, an epigenetic mechanism underlying NANOG overexpression in PDAC appeared most plausible. Our findings now provide direct evidence that NR5A2 binds to the NANOG promoter/enhancer region, thereby transcriptionally regulating its expression in colorectal CSCs.

The combination of Cmp3—a highly selective pharmacologic inhibitor of NR5A2—with standard-of-care chemotherapy exhibited robust synergistic antitumor efficacy, resulting in enhanced CSC depletion, durable tumor regression, and prolonged survival in preclinical CRC models. Notably, simultaneous administration of Cmp3 and Oxaliplatin surpassed sequential treatment in effectiveness. In patient-derived xenograft (PDX) models, the combination of oxaliplatin and Cmp3 tripled median survival and achieved complete remission in 33% of treated cases, with the most pronounced benefit observed in tumors co-expressing high levels of NR5A2 and NANOG. Importantly, Cmp3 monotherapy conferred minimal tumor control, reinforcing the concept that NR5A2 inhibition alone is insufficient to fully eradicate CSC populations. These findings underscore the therapeutic necessity of combining NR5A2 blockade with cytotoxic chemotherapy to achieve durable CSC elimination and prevent tumor relapse. Further investigations should explore combinatorial strategies, tumor microenvironment modulation, and immune system interactions.

In conclusion, NR5A2 represents a viable therapeutic target for CRC, yet optimal outcomes require its integration with chemotherapy. Treatment personalization based on molecular phenotypes may enhance response rates. Circulating tumor cells warrant assessment as non-invasive biomarkers for NR5A2/NANOG expression. Critically, our systematic integration of genomic and epigenomic regulatory layers revealed coordinated multi-omics aberrations in CRC. This approach identified molecular subtypes offering novel mechanistic insights and advancing precision diagnostics/therapeutics for CRC patients.

## Supplementary Information

Supplementary Material 1.

## Data Availability

The data that support the findings of this study are available from the corresponding author, upon reasonable request.
