# Supplementary material for: Epigenetic regulation of NR5A2 influences colorectal cancer cell stemness via a stemness-related transcription factor NANOG
Source: Cell Commun Signal. 2025 Nov 10;23:484. doi: 10.1186/s12964-025-02477-5 (PMC12604224; doi:10.1186/s12964-025-02477-5)
Supplement: Supplementary file 1 — Supplementary Material 1. [file 12964_2025_2477_MOESM1_ESM.docx]

**Supplementary Materials**

**Methods and materials**

**Profiling of DNA copy numbers, DNA methylation, and mRNA expression**

The frozen tissues from 30 cases of tumor specimens and 30 cases of the non-tumoral surrounding tissue specimens were used for DNA copy-number variation (CNV), DNA methylation (MET), and mRNA expression (EXP) profiling. Each data profile was normalized by subtracting the average values per probe of the non-tumoral tissues to represent the fold differences between the tumor and non-tumoral tissues. Then, for CNV data, gene-level DNA copy numbers in each sample were mapped with the segmented CNV values by circular binary segmentation algorithm implemented in R package library “DNAcopy.” For DNA methylation profile, probe level β-values were filtered to remove the probes located on sex chromosomes. Then, the probes residing in CpG islands-related regions including CpG islands, Shelf, and Shore regions, differentially methylated regions, and gene promoter regions including the upstream 2500 bases from TSS, 5′UTR, and first-exon regions were mapped to their corresponding genes. Then, pairwise Pearson’s correlation coefficients were calculated for each gene in the paired profiles of CNV vs. EXP and MET vs. EXP, respectively.

**Validation of the molecular subtypes using TCGA data**

Validation of the analysis results was performed using a data set of colorectal adenocarcinoma (COAD) from TCGA. Multi-layered profiles for DNA copy numbers, DNA methylation, mRNA expression, and mutations of COAD data were obtained from official TCGA data portal (https://tcga-data.nci.nih.gov). After matching sample labels in each platform, we used 364 data sets that have the matched data sets of DNA copy numbers, methylation, and mRNA expression profiles.

**Gene set enrichment and network analyses**

For each sample, the enrichment of a gene signature was calculated by applying Kolmogorov-Smirnov test. In addition, canonical gene ontology analyses for biological processes were performed using DAVID software (https://david.ncifcrf.gov). Gene networks for given gene signatures were constructed using pathway, physical, and genetic interactions that were obtained from GeneMania plugin implemented in Cytoscape software (http://www.cytoscape.org).

**RNA-seq**

Total RNA was isolated by the guanidine thiocyanate method using standard protocols. RNA Integrity Numbers were in the range of 9.2 to 10.0 when assayed on an Agilent 2100 Bioanalyzer. PolyA+ RNA fraction was extracted and randomly fragmented, converted to double-stranded cDNA. Adapter-ligated library was completed by eight cycles of PCR with Illumina PE primers. The resulting purified cDNA library was applied to an Illumina flow cell for cluster generation and sequenced on the Genome Analyzer IIx with SBS TruSeq v5 reagents by following the manufacturer's protocols. The 40-nt single-end RNA-seq sequenced reads were aligned to the human genome (GRCh37/hg19) with TopHat-2.0.4 using Bowtie 0.12.7 and Samtools 0.1.16, allowing two mismatches and five multi-hits. Transcripts assembly and estimation of their abundances were calculated with Cufflinks 1.3.0, using the human genome annotation data set Homo_sapiens.GRCh37.65 from Ensembl. Differential expression for genes across the different conditions was calculated with Cuffdiff.

**RT‑PCR**

Total RNA from the CRC tumor samples and cell lines was extracted using the RNeasy Mini kit (Qiagen, Manchester, UK) according to the manufacturer's instructions. mRNA expression levels were measured by qRT-PCR using SYBR® Premix Ex Taq™ (Takara). The cycling conditions were as follows: 95 °C for 30 s, followed by 40 cycles of 95 °C for 5 s, the annealing temperature for 5 s, and 72 °C for 30 s, and a final extension of 72 °C for 5 min. GAPDH was used as an internal control. To analyze miRNAs, first-strand cDNA synthesis and qRT-PCR were performed using Trans Script Green miRNA Two-Step qRT-PCR SuperMix (Transgen). Fold changes were calculated using the 2^-ΔΔCT^ method.

**Western blot**

Western blot analysis was performed with reference to a standard protocol. The cell extracts were collected and quantified with the BCA Protein Assay Kit (Wanlei Bio, Shenyang, China). A total of 50 μg protein was resolved by sodium dodecyl sulfate polyacrylamide gel electrophoresis and then electro-transferred to polyvinylidene fluoride membranes (Millipore, Stafford, VA, USA). Then, the membranes were blocked in 5% non-fat milk for 2 h and then incubated with primary antibodies at 4°C overnight. Secondary antibodies labeled with horseradish peroxidase (Cell Signaling Technology [CST], Danvers, MA, USA) were incubated with the membranes at room temperature for 2 h and proteins were detected using an ECL Kit (Wanlei Bio). β-actin served as the control for whole-cell lysates.

**Extreme limiting dilution analysis**

Isolated primary tumors were mechanically dissociated using a gentleMACS octo tissue dissociator with C tubes (Miltenyi Biotec). In order to consistently obtain single cell suspensions a human tumor dissociation kit (Miltenyi Biotec) was used according to manufacturer’s instruction for “tough tumors”. Briefly, tumors were cut into 2-4 mm pieces and exposed to dissociation enzymes. Following incubation at 37 °C for 30 min on an orbital shaker mechanical dissociation was carried out with the genteMACS and this process was repeated 3 times. Dissociated cells were collected after passage through a 40 μm nylon mesh filter. Live human tumor cells from xenografts were obtained by FACS on a SY3200 (Sony Biotechnology) flow cytometer after selection of DAPI- and H2KD-cells. Secondary female, 5-week old, NOD/SCID mice were inoculated with 10,000, 1,000, or 100 cells from each treatment group as described above. Tumor formation rate in secondary mice was assessed 7 weeks following implanting cells by direct palpitation and used to assess CSC frequency using the ELDA webtool (http://bioinf.wehi.edu.au/software/elda/).

Figures


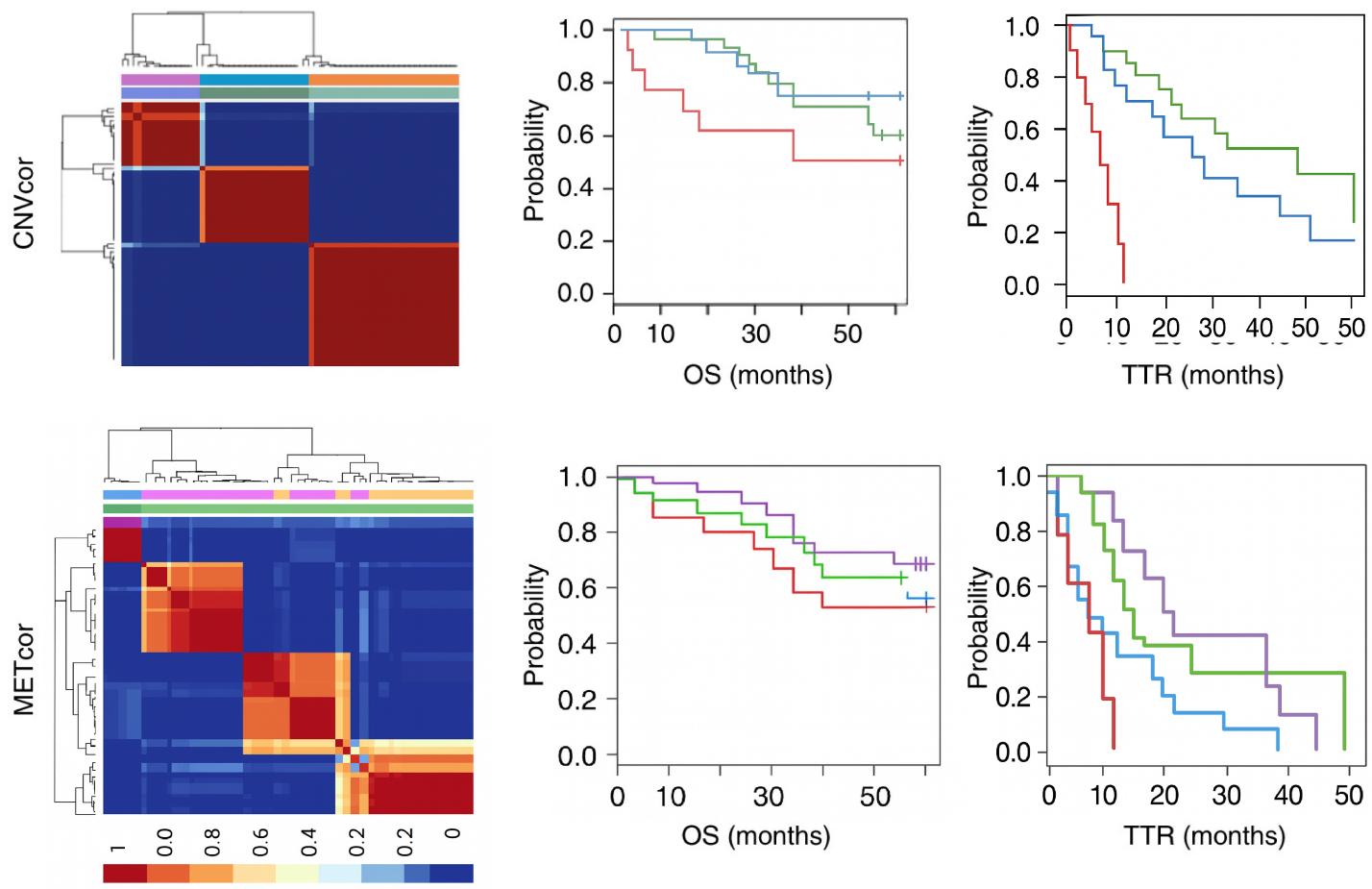


**Figure S1. DNA copy-number-correlated (CNVcor) and DNA methylation-correlated (METcor) genes predict prognostic subgroups.** Plots show the non-negative factorization (NMF) cluster results for the CNVcor in CNV data (A) and for the METcor in MET data (B), respectively. Kaplan-Meier analyses for subtypes identified by NMF clustering of the CNVcor and METcor genes are shown for overall survival (OS) and time to tumor recurrence (TTR), respectively.

**
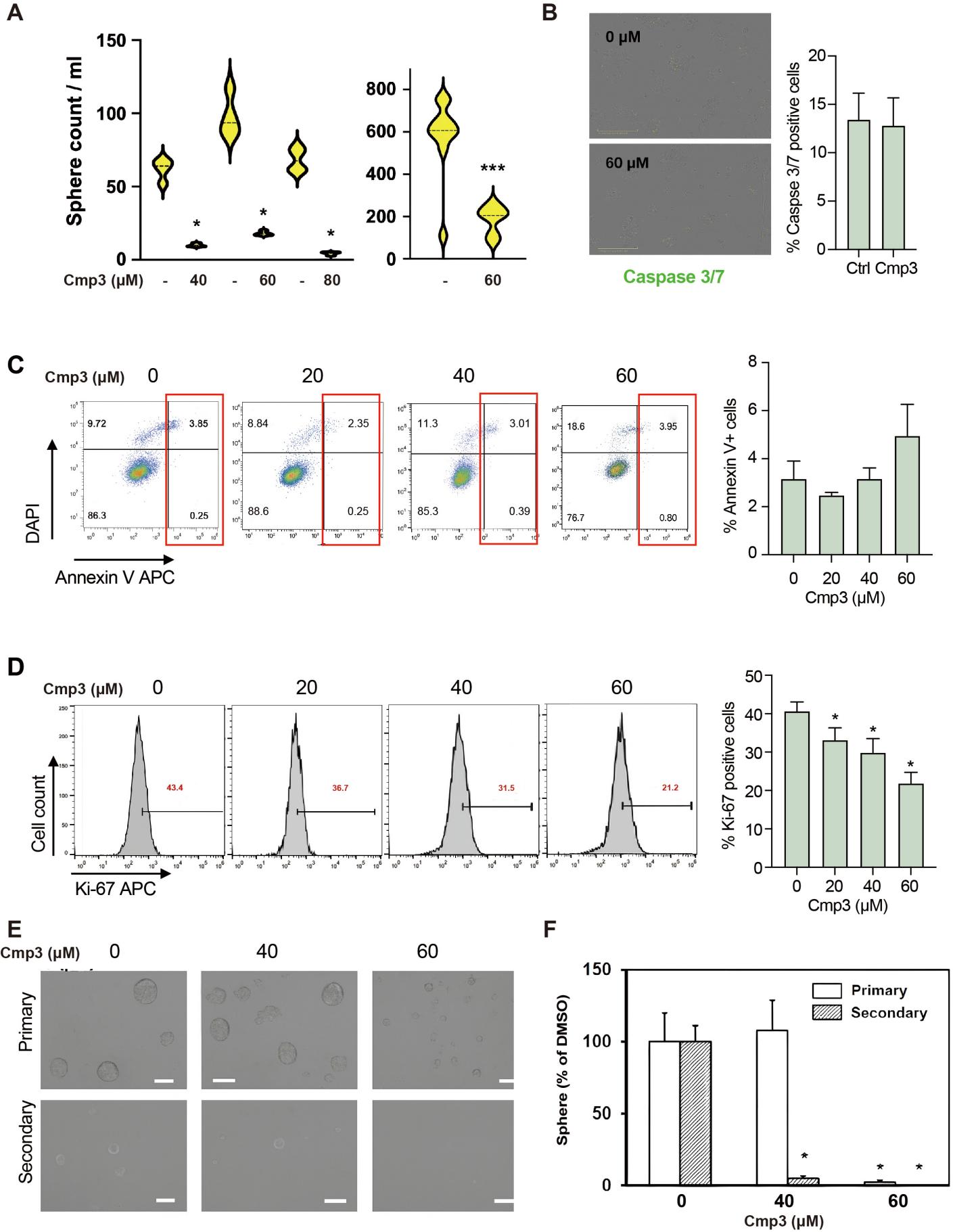
**

**Figure S2. NR5A2 regulates stemness in CRC.** (A) Quantification of primary sphere formation capacity on day 7 of Cmp3 treatment. (B) Caspase 3/7 staining was performed on cells treated with control or 60 µM Cmp3 for 72 hours. (C) Apoptosis analysis was conducted using DAPI/Annexin V flow cytometry in CRC cells treated with graded doses of Cmp3. (D) Proliferation analysis assessing the number of Ki-67+ cells was conducted in CRC cells treated with graded doses of Cmp3. Pictures of primary or secondary mammospheres were shown in (E) and inserted bars presented 100 μm. The counting results were shown in (F). * p < 0.05, ** p < 0.01, *** p < 0.001.

**
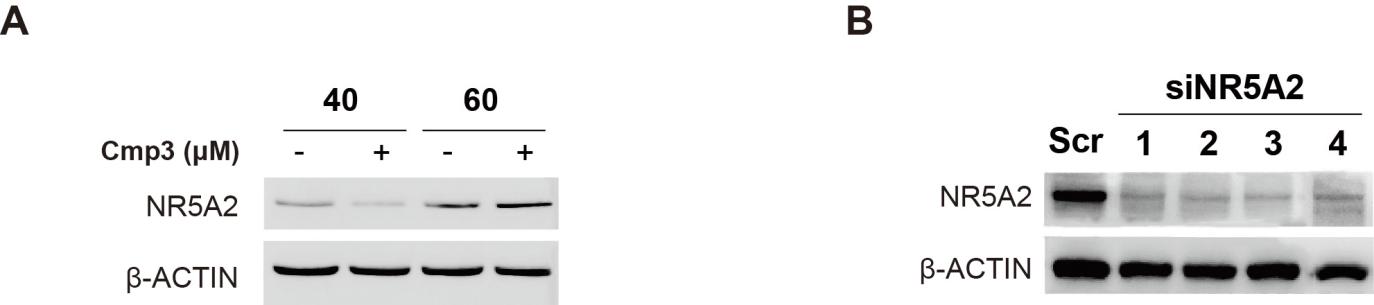
**

**Figure S3. NR5A2 regulates proliferation of differentiated CRC cells.** (A) Western blot analysis of NR5A2 protein levels at 72 hours following treatment with Cmp3 in CRC cultures. (B) Efficacy of siNR5A2 in suppressing NR5A2 protein levels was assessed by Western blot. Control (scr: scramble siRNA) and four different siNR5A2 (#1 to #4) were tested for 72 hours. β-Actin was used as the loading control.


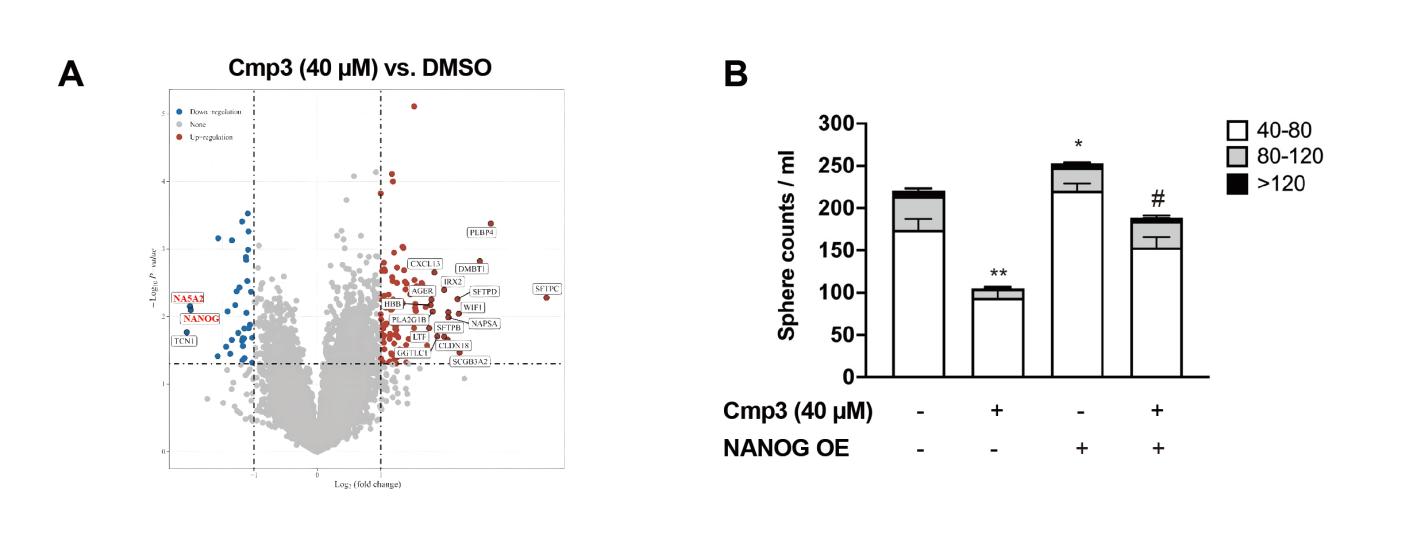


**Figure S4. NR5A2 regulates proliferation of differentiated CRC cells.** (A) RNA-seq analysis was conducted to show downregulation on NANOG target genes when NR5A2 is inhibited by Cmp3 (60 µM) for 72 hours. (B) Assessment of sphere formation capacity in cells with Scramble or NANOG OE following 72-hour treatment with DMSO (Ctrl) or Cmp3 (60 µM). Compared with DMSO+ Scramble, * p < 0.05, ** p < 0.01, *** p < 0.001. Compared with DMSO+ NANOG OE, # p < 0.05, ## p < 0.01, ### p < 0.001.


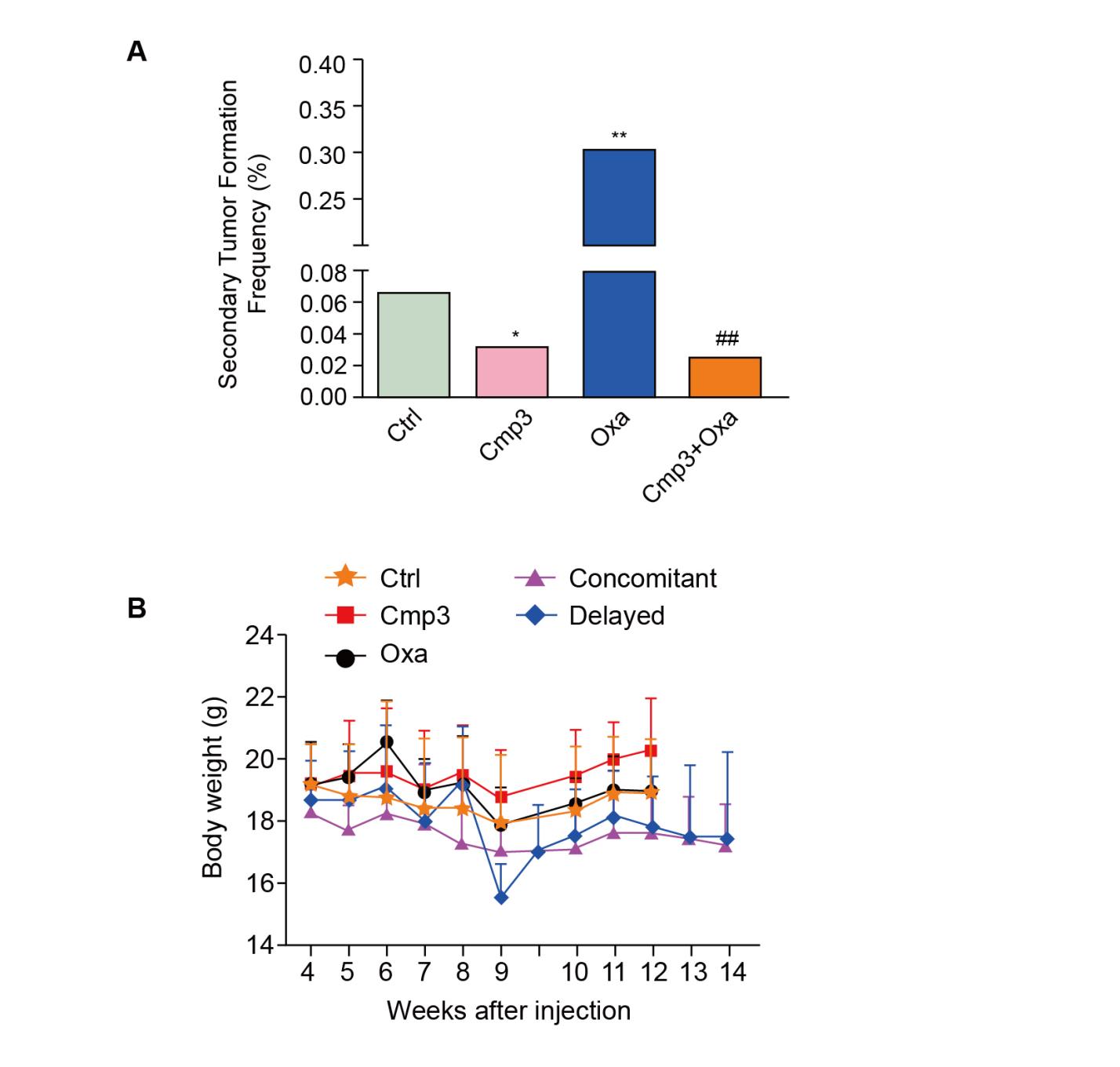


**Figure S5. NR5A2 inhibition targets CSC in vivo.** (A) The frequency of secondary tumor formation after inoculating of the isolated tumor cells from primary mice xenograft. Equal number of tumor cells isolated from primary mice xenograft were implanted in to new set of recipient mice, which did not received any therapies and were monitored for tumor initiation. The tumor initiating CSC rate was determined and plotted. (B) Body weight according to allocated treatments with two treatment cycles of 28 days each.

**Supplementary Table 1. cDNA primer sequences**

| **Target gene** | **Fw sequence** | **Rv Sequence** |
| --- | --- | --- |
| *OCT-4* | AGAACATGTGTAAGCTGCGG | GGTTCGCTTTCTCTTTCGGG |
| *NANOG* | CCTGTGATTTGTGGGCCTGA | TGCGACACTCTTCTCTGCAG |
| *KLF4* | ACCCACACAGGTGAGAAACC | ATGTGTAAGGCGAGGTGGTC |
| *SOX2* | AGAACCCCAAGATGCACAAC | CGGGGCCGGTATTTATAATC |
| *NR5A2* primer #1 | CCTTCCCAAGGCCACGAAAT | TTGAGACACAATAGGTGTAAGTCCG |
| *NR5A2* primer #2 | GGGTACCATTATGGGCTCCT | TGTCAATTTGGCAGTTCTGG |

**Supplementary Table 2 – Genomic DNA primer sequences**

| **Target gene** | **Fw sequence** | **Rv sequence** |
| --- | --- | --- |
| *nANOG* | GGCTTTGTTTGACTCCGTGT | TCCCATTGTCCCGACGTAAA |
| *NR5A2* | AGGGGTATGTCACAAGCCAA | CTCAGAAGTGGAGAGGTGCT |
| *CDKN1A* (p21) | GCTGGGATCTGATGCATGTG | ACTTCTAGCTCACCACCACC |
| Intergenic  (negative control) | CCTGCTGCCATTCACGTTAG | GACCTACTTGAGACTGGGCA |

**Supplementary Table 3 – Genetic targeting of NR5A2**

| **Target gene** | **Sequence** |
| --- | --- |
| *NR5A2* sh#1 | GCTGGACTACACAATGTGTAA |
| *NR5A2* sh#2 | CGAACTCTCGTTGATCAAA |
| *NR5A2* si#1 | ACGCATGTTAATCTATGCAA |
| *NR5A2* si#2 | ACGACAGGAGCTGATAAGCAA |
